# Patterns of renal and splanchnic sympathetic vasomotor activity in an animal model of survival to experimental sepsis

**DOI:** 10.1590/1414-431X2021e11873

**Published:** 2022-01-14

**Authors:** M.I.O. Milanez, A.M.A. Liberatore, E.E. Nishi, C.T. Bergamaschi, R.R. Campos, I.H.J. Koh

**Affiliations:** 1 Laboratório de Fisiologia Cardiovascular, Departamento de Fisiologia, Escola Paulista de Medicina, Universidade Federal de São Paulo, São Paulo, SP, Brasil; 2 Laboratório de Pesquisa Experimental, Departamento de Cirurgia, Escola Paulista de Medicina, Universidade Federal de São Paulo, São Paulo, SP, Brasil

**Keywords:** Post-sepsis, Renal sympathetic activity, Splanchnic sympathetic activity, Baroreflex control, Cardiovascular function

## Abstract

Sepsis causes long-term disability, such as immune dysfunction, neuropsychological disorders, persistent inflammation, catabolism, and immunosuppression, leading to a high risk of death in survivors, although the contributing factors of mortality are unknown. The purpose of this experimental study in rats was to examine renal (rSNA) and splanchnic (sSNA) sympathetic nerve activity, as well as baroreflex sensitivity, in acute and chronic post-sepsis periods. The rats were divided into two groups: control group with naïve Wistar rats and sepsis group with 2-mL intravenous inoculation of *Escherichia coli* at 10^8^ CFU/mL. Basal mean arterial pressure, heart rate, rSNA, sSNA, and baroreflex sensitivity were evaluated in all groups at the acute (6 h) and chronic periods (1 and 3 months). Basal rSNA and sSNA were significantly reduced in the surviving rats, as was their baroreflex sensitivity, for both pressor and hypotensive responses, and this effect lasted for up to 3 months. A single episode of sepsis in rats was enough to induce long-term alterations in renal and splanchnic sympathetic vasomotor nerve activity, representing a possible systemic event that needs to be elucidated. These findings showed that post-sepsis impairment of sympathetic vasomotor response may be one of the critical components in the inability of sepsis survivors to respond effectively to new etiological illness factors, thereby increasing their risk of post-sepsis morbidity.

## Introduction

Multiple systems, including neurological, hormonal, and metabolic pathways, are activated and/or depressed as a result of sepsis (1). The key pathogenic events in sepsis include endothelial barrier malfunction, microvascular dysfunction, fluid loss into the interstitial space, tissue edema, hypotension, and shock (2,3). Moreover, local mismatches in oxygen supply/demand due to perfusion derangements may occur (4), which may contribute to the development of acute kidney injury (AKI).

Numerous studies have demonstrated that the sympathetic nervous system (SNS) plays an important role in regulating renal vascular tone, as well as water and electrolyte reabsorption and renin secretion (5) through its dense innervation of the juxtaglomerular cells, renal tubules, and macula densa (6,7). The level of sympathetic nerve activity (SNA) is one of the determinants of renal blood filtration (RBF) and glomerular filtration rate (GFR) through a differential innervation pattern of the afferent and efferent arterioles (5), whereas reflex stimulation of renal sympathetic nerve activation with hypoxia can selectively increase or decrease glomerular capillary pressure and hence GFR by differentially activating separate populations of renal nerves (8).

Thus, to better understand the causes of septic AKI, it is necessary to improve our knowledge of the hemodynamic changes in the renal micro-vasculature, the role of increased SNA and hormonal systems, and to investigate the importance of intra-renal shunting.

Furthermore, sepsis induces long-term disability, such as immune dysfunction, neuropsychological disorders, persistent inflammation, catabolism, and immunosuppression (9,10), leading to a high risk of death in survivors (11,12). The causes of mortality, on the other hand, are still unknown. Given the global burden of sepsis, which is projected to be 48.9 million cases and 11 million fatalities (12), understanding the mechanisms underlying post-sepsis mortality appears to be critical (13).

The impairment of neurophysiological system function is common in sepsis and lasts throughout the post-sepsis phase, indicating that sepsis causes considerable neural dysfunction (14). The autonomic nervous system exerts an important reflex regulation of heart rate, cardiac output, myocardial contractility, peripheral vascular resistance, and organ blood flow distribution, as well as other adaptive mechanisms to stress (15,16).

In a number of cardiovascular diseases, including sepsis, baroreflex sensitivity is considered a valuable predictor of prognosis (17). Decreases in baroreflex sensitivity have been documented in critically ill adults with organ dysfunction and in animal models of sepsis (18,19).

In addition to long-term neurological consequences after sepsis, an interplay between severe illness and brain function may be linked to brain damage (14). Although findings point to a causal association between infection and dementia, this has yet to be established (14). A better understanding of these events should aid researchers in developing a pathophysiological explanation for sepsis survivors who have a high mortality rate, particularly in the first six months. Our hypothesis is that changes in baroreflex responses and/or basal SNA may contribute, at least in part, to the high mortality in sepsis-surviving subjects.

Therefore, the purpose of this experimental study was to examine renal (rSNA) and splanchnic (sSNA) sympathetic nerve activity and baroreflex sensitivity in acute and chronic post-sepsis periods in rats.

## Material and Methods

All experimental procedures were conducted in accordance with the guidelines of the National Institutes of Health and were approved by the Ethics in Research Committee of the Escola Paulista de Medicina, Universidade Federal de São Paulo (process Nos. 50310509/18 and 8724270715).

Female Wistar rats (200-250 g) were housed in group cages, given free access to rat chow and water, and maintained in a temperature-controlled environment (23°C) with relative humidity of 40-60% and a 12-h light/dark cycle.

### Experimental protocol

This study used two groups of animals: a control group of naive animals (n=5, 12 weeks old) and a sepsis group of animals inoculated with *E. coli.* The animals in the sepsis group were monitored during the acute phase (n=5, 6 h) and one month (n=5, 16 weeks old) and three months (n=4, 24 weeks old) after they had recovered from sepsis.

### Bacterial strain and sepsis induction

Gram-negative sepsis was induced using *E. coli* R-6 (ONT: H2 serotype). To make a nonviable bacterium inoculum, the bacteria were suspended in saline solution to a final concentration of 10^8^ colony forming units (CFU)/mL and then formalin solution (0.5%) was added. After 48 h, the formalin solution was washed away with sterile saline, and the nonviable bacteria inoculum (2 mL of 10^8^ CFU/mL per animal) was slowly injected into the jugular vein under general anesthesia (3% isoflurane). A previous study showed that sepsis challenge with either live or dead bacteria prompted similar clinical outcomes (20). We also chose this strategy to avoid antibiotic treatment after sepsis and for the safety of the researchers. This experimental sepsis model causes 50-60% mortality between 12 and 27 h.

### Recording of mean arterial pressure and heart rate

Rats under ketamine and xylazine (Vetbrands, Brazil) anesthesia (80 and 10 mg/kg, respectively) had the femoral artery and vein catheterized for blood pressure (BP) recordings and drug administration, respectively. After surgical recovery (approximately 24 h), baseline pulsatile BP, mean arterial pressure (MAP), and heart rate (HR) were recorded in conscious rats (PowerLab, ADInstruments, Australia) for approximately 20 min.

### rSNA and sSNA analysis in anesthetized rats

After cardiovascular baseline recordings (MAP and HR), urethane was administered intravenously (1.4 g/kg, Sigma Aldrich, USA). Then, the left renal and splanchnic nerves were retroperitoneally exposed and placed on bipolar silver electrodes. Once the conditions for nerve recording were established, the nerve and electrode were covered with paraffin oil. The signals from the nerves were displayed on a TDS 220 oscilloscope (Tektronix, USA), and nerve activity was amplified (gain 20 K, NeuroLog, Digitimer, UK), filtered with a bandpass filter (100-1000 Hz), and collected for display and subsequent analysis using a PowerLab data acquisition system (ADInstruments).

As previously described (21), the baroreceptor sensitivity was determined by linear regression for sympathoinhibitory and sympathoexcitatory responses after ramp infusion of phenylephrine (100 µg/mL) or sodium nitroprusside (200 µg/mL), respectively, over 60 s (0.1 mL). The inhibitory and excitatory reflex changes in rSNA or sSNA were measured for every 5 mmHg change in MAP (from 5 to 40 mmHg). The regression coefficient (slope) analysis represents the baroreflex gain (ΔrSNA/ΔMAP or ΔsSNA/ΔMAP) for each individual animal and is reported as spikes/s per mmHg. The number of spikes of identified action potentials in the raw and filtered recordings was evaluated using the Lab Chart/Spike histogram tool (LabChart 7 software, ADInstruments). For that, at the end of the experiments, the background noise level of the recorded nerves was determined after hexamethonium bromide intravenous administration (30 mg/kg, Sigma Aldrich); the number of spikes was counted by spikes per second (spikes/s) that exceeded the background noise threshold. Thus, the basal rSNA or sSNA is reported as spikes/s (1-s bins) over a period of 120 s, as previously reported (22,23).

### Data analysis

Data are reported as means±SE. The normality of data was tested by the Kolmogorov-Smirnov test. The data was evaluated by one-way ANOVA followed by the Newman-Keuls *post hoc* test (GraphPad Prism 5, USA). The level of statistical significance was defined as P≤0.05.

## Results

### Basal SNA and macrocirculation

During the acute phase of sepsis (6 h), spike variations were seen in both territories but in the opposite direction, with an rSNA reduction indicating a response to renal vasodilation and an increased sSNA indicating a vasoconstriction in the intestinal region, but neither was statistically significant when compared to their control value. It was, however, accompanied by an increase in HR, possibly to preserve MAP stability (Table 1). In comparison, sSNA decreased in the 1-month group, whereas both rSNA and sSNA decreased significantly in the 3-month group. The animals that survived sepsis did not differ from the control group in terms of MAP or HR.

**Table 1:** Comparison of renal and splanchnic sympathetic nerve activity (rSNA and sSNA, respectively), mean arterial pressure (MAP), and heart rate (HR) values between the control and sepsis groups at different time-points.

	Control (n=5)	6 h (n=5)	1 month (n=5)	3 months (n=4)	F	R^2^
rSNA (spikes/s)	107±11	91±10	76±13	58±12*	3.1 (P<0.05)	0.38
sSNA (spikes/s)	67±4	80±6	38±3*	45±8*	11.1 (P<0.01)	0.67
MAP (mmHg)	110±4	102±8	106±7	106±9	0.09 (P=0.9)	0.01
HR (bpm)	377±17	412±11	361±19	363±25	1.7 (P=0.2)	0.25

Data are reported as means±SE. *P≤0.05 compared to control (one-way ANOVA, followed by Newman-Keuls *post hoc* test). F: F ratio; R^2^: correlation coefficient.

**Figure 1:**
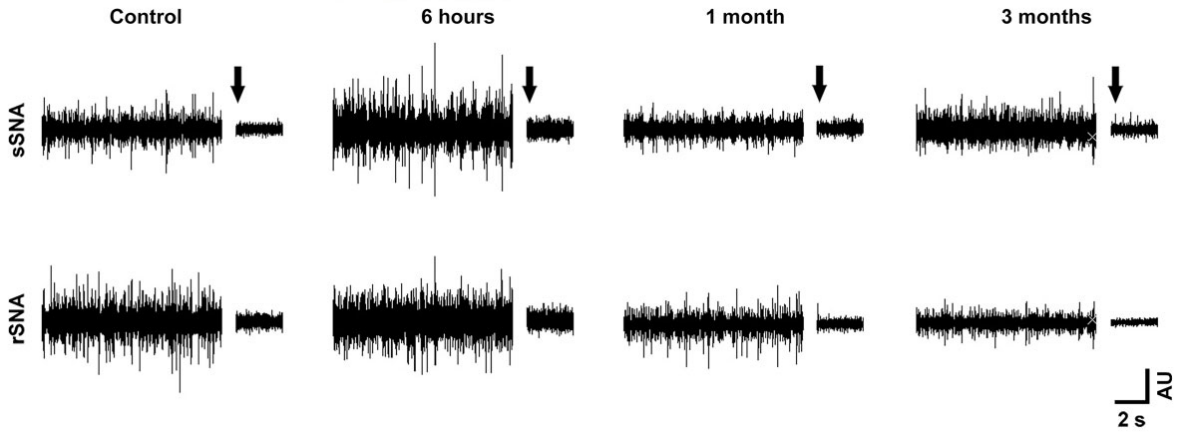
Diagram representing the basal renal/splanchnic sympathetic nerve activity in control rats and in rats 6 h, 1 month, and 3 months after sepsis. Sepsis surviving rats exhibited a lower level of sympathetic vasomotor activity. The arrow indicates the background noise threshold triggered by intravenous hexamethonium. rSNA: renal sympathetic nerve activity; sSNA: splanchnic sympathetic nerve activity.

The overall data in the first three months after sepsis showed profound changes in sympathetic vasomotor activity. We then evaluated the functional response of baroreceptors during hyper- and hypotension states induced by vasoactive drugs. Figure 1 shows a representative diagram of the basal rSNA and sSNA of the groups assessed in this study.

### Regulation of SNA by baroreceptors

The results of the reflex sensitivity of sympathetic nerve responses to gradual hypotension and hypertension situations (Δ40 mmHg) revealed an altered control in the acute period of sepsis (6 h) and 1 and 3 months after sepsis recovery. Furthermore, the baroreceptor response sensitivity varied between the two regions as well as between hypotension and hypertension states.

**Figure 2:**
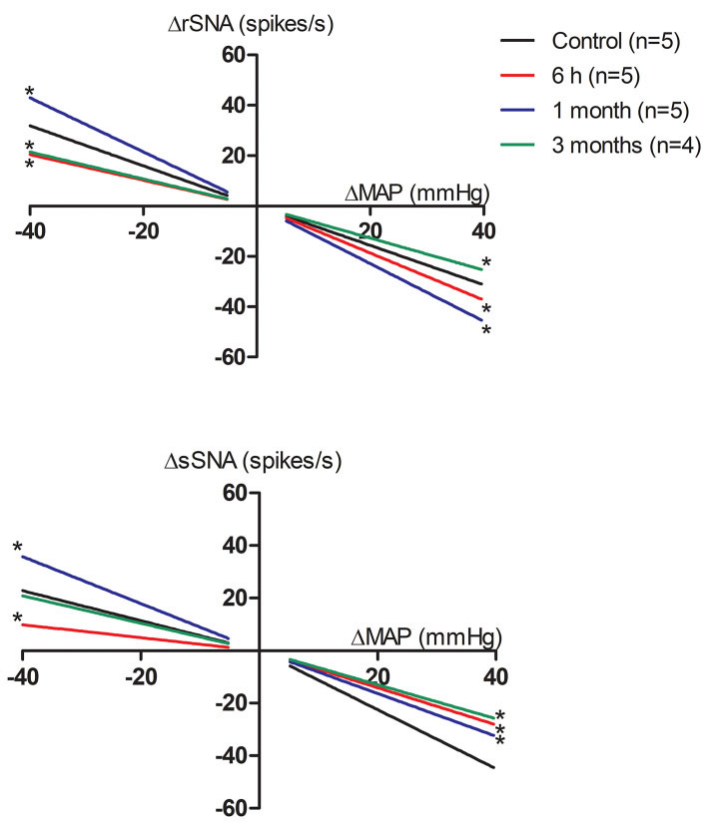
Analysis of the renal and splanchnic sympathetic reflex responses at 6 h, 1 month, and 3 months post-sepsis under vasoactive drug-induced hypotension and hypertension states. rSNA: renal sympathetic nerve activity; sSNA: splanchnic sympathetic nerve activity; MAP: mean arterial pressure. Data are reported as means±SE. *P<0.05 compared with control (one-way ANOVA followed by the Newman-Keuls *post hoc* test).

In the 6-h group, a reduction was found in the sensitivity of the arterial baroreceptor reflex to the sSNA, as well as impairment of sympathoexcitatory rSNA reflex responses. However, the rSNA sympathoinhibitory reflex response was increased in the 6-h group (Figure 2).

In the 1-month group, the renal sympathoexcitatory and sympathoinhibitory reflex responses were significantly increased compared with control rats. Conversely, the splanchnic sympathoexcitatory response was increased and the sympathoinhibitory response was reduced in 1-month rats compared with control rats. However, the opposite was found in the 3-month group, and the renal sympathoexcitatory and sympathoinhibitory responses were significantly reduced compared with control rats. The control of sSNA by arterial baroreceptors in the 3-month group was reduced only for sympathoinhibitory reflex responses (Figure 2). The quantitative values of the baroreflex sensitivity (slopes) are shown in Table 2.

**Table 2:** Baroreflex assessed by changes in renal and splanchnic sympathetic vasomotor activity (rSNA and sSNA, respectively) in response to mean arterial pressure variations induced by systemic administration of vasoactive drugs in the control and sepsis groups at different time-points.

	rSNA reflex increase	rSNA reflex decrease	sSNA reflex increase	sSNA reflex decrease
Control (n=5)	-0.79±0.08	-0.78±0.08	-0.57±0.06	-1.12±0.07
6 h (n=5)	-0.51±0.05*	-0.93±0.06*	-0.24±0.02*	-0.70±0.03*
1 month (n=5)	-1.07±0.07*	-1.14±0.05*	-0.89±0.07*	-0.81±0.06*
3 months (n=4)	-0.53±0.03*	-0.63±0.07*	-0.52±0.09	-0.64±0.04*
F	75.45 (P<0.0001)	42.52 (P<0.0001)	80.21 (P<0.0001)	63 (P<0.0001)
R^2^	0.93	0.89	0.94	0.92

Data are reported as means±SE (spikes/s per mmHg). *P<0.05 compared to control (one-way ANOVA, followed by Newman-Keuls *post hoc* test). F: F ratio; R^2^: correlation coefficient.

## Discussion

This study reports changes in renal and splanchnic SNA in rats that survived an episode of sepsis, showing that, in addition to a change in the first hour after sepsis induction, this phenomenon persisted in surviving animals, at least until the third month after the episode. Despite normal baseline values of MAP and HR, basal rSNA and sSNA were significantly reduced in surviving rats, as well as the baroreflex sensitivity, for both pressor responses and hypotensive responses.

Our data showed that both rSNA and sSNA basal values declined as the post-sepsis period progressed, with no evidence of improvement after 3 months, suggesting a long-term central and/or peripheral alteration leading to arterial baroreceptor and/or SNA dysfunction in surviving rats.

The arterial baroreceptor reflex sensitivity of sSNA increased after 6 h of sepsis and was reduced in rSNA. This fact could point to a redistributive mechanism of blood volume in these regions with the goal of lowering splanchnic flow while maintaining renal perfusion. The experimental sepsis model used is characterized by an initial hyperdynamic phase followed by a moderate and transient hypodynamic phase around 6-8 h after sepsis induction, and with a 50 to 60 percent mortality rate up to 27 h (20). In this acute phase of sepsis, data suggest that the role of SNA in blood redistribution is still preserved, but this hypothesis does not apply to the findings after 1 and 3 months of sepsis induction, when the surviving animals presented normal MAP and HR and a healthy appearance.

The videomicroscopy of the microcirculatory hemodynamics of abdominal organs (20) and macro-hemodynamic and systemic repercussions in surviving animals have been previously examined using this model (24). Shortly after sepsis, at six hours, the kidney cortex microcirculation was severally dysfunctional by the reduction of peritubular microvessels and congestion of perfused microvessels. In addition, the edema of convoluted tubules in epithelial cells compressed the tubule lumen and the peritubular microvessels (25). The hemodynamics of microcirculation in sepsis survivors improved gradually but partially over time. Moreover, the significantly decreased blood flow in renal vessels 3 months after sepsis suggested a kidney hypoperfusion state with microcirculatory dysfunction.

Functional injury may result from regional perfusion mismatches leading to altered perfusion of critical sites such as the glomerulus, which can alter renal function, and/or local ischemia. Such changes in renal blood flow (RBF) distribution may occur as a consequence of alterations in the levels of circulating and local hormones, activation of the SNS, micro-vascular alterations with local areas of vasodilatation and vasoconstriction, opening of vascular shunts, development of micro-thrombi, or due to interstitial edema slowing oxygen diffusion, or most likely a variable combination of these mechanisms (26).

In sepsis, local mismatches in oxygen supply/demand due to perfusion derangements may occur (4) and may contribute to the development of AKI. Sepsis causes a decrease in total RBF due to renal artery vasoconstriction and/or decreased systemic delivery of blood to vital organs, and this event, in turn, causes renal ischemia and tubular cell death with loss of function. As a consequence of such ischemia, acute tubular necrosis is believed to occur (26).

About one-third of critically ill patients with AKI develop persistently decreased kidney function, known as acute kidney disease, which may progress to chronic kidney disease. Although sepsis is the most common cause of AKI, little is known about sepsis-associated acute kidney disease (27).

Our findings indicated that rSNA and sSNA decrease significantly in the post-sepsis period, potentially influencing their intra-organ role. Whether this indicates less pro-inflammatory activity was not examined, although it appears to be consistent with post-sepsis immunosuppression, as referred by others (9).

Clinical investigations have revealed that patients who survive sepsis can have complications, including cardiovascular disease, pneumonia, and AKI, among other serious disorders that can lead to death, primarily owing to infections (9,10,12,13,28).

Several risk factors for the high mortality in these patients have been reported (9,10,12), but SNA dysfunction in the post-sepsis phase has not been investigated. Many sepsis survivors exhibit cognitive changes, which have been linked to a complex interplay involving ischemia, inflammation, oxidative stress, microglial activation, and blood-brain barrier disruption (14).

The control of blood flow by the autonomic nervous system in each region is determined by perfusion pressure and flow resistance (29). In a feedback control system that regulates BP, autonomic nerves and circulating hormones function as effector mechanisms.

In different parts of the body, the activity of sympathetic vasomotor nerves, levels of circulating vasoactive hormones, and local variables such as endothelium and metabolite demand influence vascular resistance (30).

In the present study, renal and splanchnic reflex responses to intravenous phenylephrine or nitroprusside were studied to verify the baroreflex function in the acute phase of sepsis, as well as in sepsis-surviving animals. Compared with sSNA, rSNA displayed a higher reflex response to a decrease in BP and a decreased response to an increase in BP in naive animals, indicating that there is a differential regional regulation mediated by sympathetic nerves in response to BP changes. This fact can be attributed to the control of the regional distribution of blood flow and peripheral vascular resistance to maintain the homeostasis of the cardiovascular system.

Six hours after sepsis induction, the reflex response of rSNA and sSNA to BP decrease was lower than in control animals, suggesting an impairment of the sympathoexcitatory responses to renal and splanchnic regions secondary to systemic inflammation. Indeed, in the acute phase of sepsis, an experimental study on endotoxemia in healthy adults found that the sympathetic control of BP via the vascular pathway and the baroreflex system is impaired, resulting in a diminished ability to respond to induced hypotension (31).

The mechanisms that cause changes in cardiovascular reflexes in endotoxemia are not completely understood. The nucleus of the solitary tract (NTS) receives afferent inputs from aortic and carotid baroreceptors and then integrates and transmits the information to other sites in the medulla, triggering a sympathetic or parasympathetic response depending on BP changes (29). As a result, after endotoxemia, alterations in NTS neurotransmission can lead to abnormalities in baroreceptor function. Amorim et al. (32) recently described that baroreflex dysfunction after lipopolysaccharide administration may be strongly associated with an inflammatory process in the NTS. A role for NTS neurotransmission or inflammatory state in baroreflex dysfunction in the post-sepsis period remains to be determined.

Overall, the current findings showed that a single sepsis exposure caused long-term changes in the basal and reflex control of SNA. It is unknown whether these were caused by central, peripheral, or both nervous system components, or due to the association with morphological, hormonal, immunological, and local or systemic microcirculatory alterations. However, our data indicated that post-sepsis impairment of the sympathetic vasomotor activity responses may be a contributing factor to an inadequate physiological response to new etiological illness factors that may be confronted by sepsis survivors.

Although the findings from experimental animal research may not be entirely applicable to the etiopathogenesis of illnesses in human sepsis survivors, experimental studies do not face the ethical concerns about invasive interventions on humans, allowing new hypotheses to contribute to the understanding of post-sepsis pathophysiology. Moreover, with respect to sex differences, previous studies have shown that female rodents have an enhanced immunological response, resulting in better survival after injury than male rats; this difference has been attributed to hormonal aspects of each sex (33). Thus, we cannot assert that the findings described here can be reproduced with exact similarity in male rats; such phenomenon should be investigated.

In addition, it is known that there is a strong baroreceptor influence on the occurrence of neural spikes in sympathetic nerves (34,35), a fact that led us to quantify the renal and splanchnic baroreflex responses in terms of spikes/s. However, we believe that the evaluation of the characteristics of sympathetic bursts, such as their amplitude and occurrence, may reveal different aspects of the central involvement in sympathetic reflex control during and after sepsis. In fact, burst amplitude may reflect the recruitment of fibers activated within each discharge (36). Moreover, this study was limited to evaluating the sympathetic arm of the baroreflex to the kidneys and splanchnic regions and, considering that the cardiac component of the baroreflex may not behave in a redundant manner (37,38), further studies are needed to clarify the functionality of the cardiac arm of the baroreflex in sepsis-surviving models. Given our experimental conditions, the interpretations of cardiac baroreflex in anesthetized animals in this manuscript may be premature because urethane severely impairs the cardiac baroreflex while maintaining the characteristics of the sympathetic arm of the baroreflex (39).

More research is needed to determine whether SNA changes after sepsis are caused by central and/or peripheral nervous system injury, as well as the temporal length. Furthermore, determining how these alterations may affect other body compartments seems to be critical.
